# Broadband single-phase hyperbolic elastic metamaterials for super-resolution imaging

**DOI:** 10.1038/s41598-018-20579-8

**Published:** 2018-02-02

**Authors:** Hao-Wen Dong, Sheng-Dong Zhao, Yue-Sheng Wang, Chuanzeng Zhang

**Affiliations:** 1 Department of Applied Mechanics, University of Science and Technology Beijing, Beijing, 100083 China; 2 Institute of Engineering Mechanics, Beijing Jiaotong University, Beijing, 100044 China; 3 Department of Civil Engineering, University of Siegen, D-57068 Siegen, Germany

## Abstract

Hyperbolic metamaterials, the highly anisotropic subwavelength media, immensely widen the engineering feasibilities for wave manipulation. However, limited by the empirical structural topologies, the reported hyperbolic elastic metamaterials (HEMMs) suffer from the limitations of the relatively narrow frequency width, inflexible adjustable operating subwavelength scale and difficulty to further improve the imaging resolution. Here, we show an inverse-design strategy for HEMMs by topology optimization. We design broadband single-phase HEMMs supporting multipolar resonances at different prescribed deep-subwavelength scales, and demonstrate the super-resolution imaging for longitudinal waves. Benefiting from the extreme enhancement of the evanescent waves, an optimized HEMM at an ultra-low frequency can yield an imaging resolution of ~*λ*/64, representing the record in the field of elastic metamaterials. The present research provides a novel and general design methodology for exploring the HEMMs with unrevealed mechanisms and guides the ultrasonography and general biomedical applications.

## Introduction

Metamaterials are artificial subwavelength composite materials or structures, which provide many encouraging opportunities to modulate and control the wave propagation with the extraordinary physical properties. In recent years, due to their strongly anisotropic dispersion, hyperbolic metamaterials (HMMs) with hyperbolic dispersions^1^ as one of the most important types of the metamaterials have attracted special attention and evidenced many promising applications, including negative refraction^2–4^, enhanced superlensing effects^5–12^, backward waves^7^, strong thermal emission^13–16^, sensing^17^, Purcell factor enhancement^2,18^, improved absorption^19–21^, heightened conductivity^22^ and intensified spontaneous emission^2,23–26^. In particular, compared with the double-negative metamaterials, the HMMs just need to satisfy the criterion of constraining the particle motion in one or two principal directions^2^. The concept of HMMs has been applied to engineering materials for better controlling the electromagnetic waves^2,5,6,12–27^ and acoustic waves^3,4,7,9^. Unlike their electromagnetic^5^ and acoustic^28^ counterparts, elastic metamaterials (EMMs)^29–34^ involve more material parameters and support both longitudinal and transverse wave modes. They can offer more possibilities to explore unusual physical phenomena^29–34^ beyond natural materials. Therefore, by combing the characteristics of the HMMs and EMMs, it is more challenging to construct hyperbolic elastic materials (HEMMs)^8,10,11,35^ with a set of desired properties.

Over the past few years, several research groups have focused on the HEMMs and proposed different microstructural topologies^8,10,11,35^. The superlenging capacities of the HEMMs have been numerically^8,10,11^ and experimentally demonstrated^8,11^. Two different mechanisms^8,10,11,35^ have been shown to be responsible for the hyperbolic dispersions. A coiling-up metamaterial possessing different deformations along two principal directions was reported for the superior imaging resolution which breaks the diffraction limit^8^. Then, an elastic hyperlens was designed based on the microstructure with anisotropic mass densities^10,11^. In fact, for elastic media, it is generally easier to acquire a low-frequency bandgap through anisotropic mass densities than through distinct deformation mechanisms. However, a systematical design of the anisotropic mass densities for the hyperbolic dispersion is still lacking. Moreover, the previously reported HEMMs show the following limitations: (1) The operating frequency range is relatively narrow, which demands further improvements in widening the frequency and wave-vector ranges for hyperbolic eigenfrequency curves (EFCs). (2) The HEMMs at ultra-low frequencies have not yet been reported. (3) No simple and controllable method is available to design HEMMs at different subwavelength scales. (4) The imaging resolution of the elastic hyperlens has yet to be improved. We believe that the super-resolution (i.e., much smaller than the diffraction limit) ability of the HEMMs may lead to great challenges for metamaterial engineering. However, the conventional manual and intuitive designs are unable to overcome the above limitations and thus make the design of the HEMMs more challenging. Therefore, a systematic methodology is necessary for searching the high-performance microstructural topologies exhibiting the hyperbolic dispersion in modulating the elastic subwavelength waves.

In this paper, based on the topology optimization^12,33,35–37^ and effective medium theory^29,31^, we present a sophisticated design strategy to realize two-dimensional (2D) broadband single-phase HEMMs with negative effective mass densities along one principal direction. We show the similar geometrical features of the optimized HEMMs and reveal their special multipolar resonance mechanisms and controlled vibration along the wave propagating direction. All optimized HEMMs presented in this paper are proved to support the subwavelength imaging. In particular, we demonstrate that a single-phase metamaterial with suitable constraints can exhibit the hyperbolic dispersion in the ultra-low frequency range, implying the comparable capacity of manipulating elastic waves as in the multi-phase local resonance metamaterials. As a result, the longitudinal waves can propagate only along the desired direction within the HEMMs. Furthermore, our optimized HEMMs can persistently and intensely enhance the transmission of the evanescent waves over the largest wave vector range. In this way, we obtain a super-high, or almost ultimate, imaging resolution (~*λ*/64) which represents the record in the field of EMMs for longitudinal waves.

## Results

### Methodology

**Figure 1:**
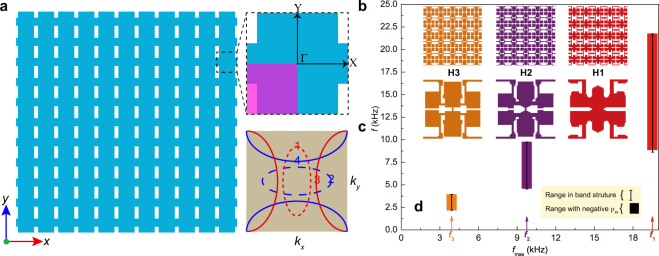
Schematic illustration of an anisotropic metamaterial and the topology-optimized results. (**a**) Metamaterial with periodic microstructures. (**b**) Unit-cell with an orthogonal symmetry. (**c**) Possible EFCs for an anisotropic metamaterial. The principal directions (ΓX and ΓY) of the first Brillouin zone are shown in (**b**) as well. The unit-cell surrounded by the dashed lines in (**b**) is taken as the design domain with the lower left quarter showing the reduced design region in optimization. (**d**) Microstructures for different target frequencies (*f*_max_ = *f*_1_, *f*_2_ and *f*_3_). Note that the optimization parameters for H1, H2 and H3 are selected as (*f*_max_ = *f*_1_ = 19.5 kHz, *δ*_E_ = 0.1), (*f*_max_ = *f*_2_ = 9.75 kHz, *δ*_E_ = 0.1) and (*f*_max_ = *f*_3_ = 3.904 kHz, *δ*_E_ = 0.05) respectively. Their operating wavelengths are *λ*_1_ = 10*a*, *λ*_2_ = 20*a* and *λ*_3_ = 50*a*, respectively. The solid rectangles represent the lower and upper frequencies of the range with a negative *ρ*_*xx*_. The line bars show the relevant frequency ranges in the band structures. Their corresponding 5 × 5 lattice structures are also shown.

To obtain the hyperbolic dispersion, we have to construct the microstructure with anisotropic mass densities^10^ or elastic moduli^11^. In this paper, we consider a single-phase metamaterial with an orthotropic symmetry in a square lattice, as shown in Fig. 1(a). Changing the microstructural topology in Fig. 1(b) will induce the possible resonances, leading to an anisotropic dispersion for a certain energy band. As evidenced in the recent work on HEMMs^10^, if the dominated mechanism is the anisotropic mass density, the eigenfrequency curves (EFCs) will be elliptical or hyperbolic, as displayed in Fig. 1(c). Especially, when *ρ*_*yy*_ > *ρ*_*xx*_ > 0 or *ρ*_*xx*_ > *ρ*_*yy*_ > 0, the curve may take the elliptical shape 1 or 2. However, if the anisotropy becomes stronger, the hyperbolic shape 3 or 4 is possible to occur for the EMM with a single negative mass density *ρ*_*yy*_ < 0 < *ρ*_*xx*_ or *ρ*_*xx*_ < 0 < *ρ*_*yy*_, like its electromagnetic and acoustic counterparts^2,9^. To systematically achieve hyperbolic EFCs in a robust way, we apply the topology optimization in this paper to design the unit-cell microstructure with a single negative mass density by considering the design domain illustrated in Fig. 1(b).

It was reported that a negative mass density can be usually realized by dipolar resonances, whereas a negative bulk modulus and a negative shear modulus can be induced by monopolar and quadrupolar resonances, respectively^29–31,33^. Although the topology optimization may yield complex microstructures beyond the existing resonance mechanisms, the involved varying characteristics of the effective parameters are similar for a metamaterial with either negative mass density or negative elastic modulus. That is, the value of the effective material parameter reaches the infinity at the resonant frequency, and then gradually decreases with the frequency away from the resonance^29–31,33^. For simplicity, it is effective to adopt the discrete responses at a certain number of frequencies to define the effective performance. To this end, we select some sampling frequencies distributed uniformly in a target frequency range (*f*_min_, *f*_max_). Generally, the essential condition for obtaining a negative mass density is to excite a suitable resonance^33^. Then the negative range can be expanded if the resonant frequency is pushed down. For the same frequency range of interest, the decrease of the resonant frequency will result in a smaller minimal positive value. Consequently, the driving force for the sufficiently wide negative range to a low frequency is to increase the ratio between the positive maximal value to the positive minimal value at all sampling frequencies. For the broadband negative mass density along the *x*-direction, we propose the topology optimization formulation within (*f*_min_, *f*_max_) as given in Table 1.

**Table 1:** Optimization objective functions and constraints.

Maximize: $SN={N}-\frac{\mathop{{\rm{\min }}}\limits_{\forall m\subset (1,\,2,\cdots ,M)}({\rho }_{xx}^{m+})}{\mathop{{\rm{\max }}}\limits_{\forall m\subset (1,\,2,\cdots ,M)}({\rho }_{xx}^{m+})}\,(1{\rm{a}})$						
**Subject to**:						
(1b)	(1c)	(1d)	(1e)	(1f)	(1g)	(1h)
$\mathop{{\rm{\min }}}\limits_{\forall i}({\rho }_{yy}^{i}) > 0$	$\mathop{{\rm{\min }}}\limits_{\forall i}({E}_{xx}^{i}) > 0$	$\mathop{{\rm{\min }}}\limits_{\forall i}(\frac{{E}_{yy}^{i}}{{E}_{xx}^{i}}) > 1.0$	$\mathop{{\rm{\min }}}\limits_{\forall i}(\frac{{E}_{xy}^{i}}{{E}_{yy}^{i}})\ge {\delta }_{{\rm{E}}}$	$\mathop{{\rm{\max }}}\limits_{\forall i}(\frac{\sum |{F}_{x}^{i}|}{\sum |{F}_{y}^{i}|})\le {\delta }_{{\rm{F}}}$	$\frac{\mathop{{\rm{\max }}}\limits_{\forall i}({\rho }_{yy}^{i})}{\mathop{{\rm{\min }}}\limits_{\forall i}({\rho }_{yy}^{i})}\le {\delta }_{\rho }$	$\mathop{{\rm{\min }}}\limits_{\varphi }(e)\ge e\ast$

Here, *ρ*_*xx*_ (*ρ*_*yy*_) and *E*_*xx*_ (*E*_*yy*_) are the effective mass density and effective elastic modulus along the *x*− (*y*−) direction, respectively; ${\rho }_{xx}^{+}$ represents the special array composed of the positive values; *E*_*xy*_ is the coupling modulus; *F*_*x*_ (*F*_*y*_) is the magnitude of the reaction force along the *x*− (*y*−) direction for calculating *ρ*_*yy*_; Σ stands for the integration over the upper and lower boundaries of the unit-cell; *e* is the array composed of the width of each solid connection; and *δ*_E_, *δ*_F_, *δ*_*ρ*_ and e^*^ are the self-defined optimization parameters; *M* is the number of the sampling frequencies; *SN* denotes the value of the objective function value; *N* is the number of the sampling frequencies where *ρ*_*xx*_ is negative; *m* (*m* ≤ *M*) is the serial number of the frequency where positive *ρ*_*xx*_ remains; and *i* (*i* = 1, 2…*M*) is the serial number of the calculated frequencies. The constraints (1b)-(1d) are introduced to ensure the emergence of the longitudinal wave motion in the *y*-direction. The constraint (1e) is introduced to control the exclusive longitudinal wave motion along the *y*-direction. The constraint (1 f) is used for ensuring a purely translational motion in the *y*-direction and deleting the pronounced local rotations^29^. The constraint (1 g) is employed to guarantee the strong anisotropy of the effective mass densities. Finally, the constraint (1 h) is utilized to make the structure sufficiently stiff and manufacturable. Our numerical tests show that *M* = 11 can effectively describe the continuous dynamic properties over a wide frequency spectrum. More sampling frequencies will result in a higher computational cost but not alter the optimized results essentially. For all designs with a negative *ρ*_*xx*_, we take *δ*_F_ = 0.2, *δ*_ρ_ = 1.37 and e^*^ = 0.001 m based on the numerical tests. The genetic algorithm (GA) is adopted^33^ to achieve the optimized HEMM for a given frequency range (*f*_min_, *f*_max_). The kernel of the present design method is the fact that the GA generates various microstructural topologies whose effective material parameters are extracted by the effective medium theory to hunt for better objective properties. More details on the determination of the effective parameters and the descriptions of the objective function and constraints are presented in the Supplemental Material^38^.

### Optimized metamaterials

We consider the design of a square-latticed (lattice constant *a* = 0.03 m) perforated single-phase metallic structure made of the stainless steel^10,31,33^ with the mass density *ρ* = 7850 kg m^−3^, the Young’s modulus *E* = 200 GPa and the Poisson’s ratio *υ* = 0.3. By employing the topology optimization, we construct some novel microstructural topologies which are difficult to conceive through a conventional intuition. These distinct HEMMs show outstanding frequency bandwidths and profoundly reveal some exotic mechanisms for the hyperbolic dispersion. Figure 1(d) shows the optimized microstructures H1, H2 and H3 at the different wavelength scales of *λ*_1_ = 10*a* (*f*_max_ = 19.5 kHz), *λ*_2_ = 20*a* (*f*_max_ = 9.75 kHz) and *λ*_3_ = 50*a* (*f*_max_ = 3.904 kHz), respectively. The evolutionary history and the convergence of the topology optimization strategy for H3 are described in the Supplemental Material^38^. It is noted here that the optimized solutions can certainly produce a certain bandwidth of the negative *ρ*_*xx*_ below *f*_max_. Since we are intended to obtain a sufficiently wide hyperbolic range at low frequencies as much as possible, *f*_min_ for all optimization cases is selected as 0.5 Hz. We use different *δ*_E_ to appropriately confine the coupling modulus to ensure the emergence of the negative *ρ*_*xx*_ in the given search space, especially for the cases at ultra-low frequencies. It is seen from Fig. 1(d) that the three ranges of the negative *ρ*_*xx*_ are nearly consistent with the relevant frequency ranges in the band structures. The negative ranges with the mid-frequencies of 15.3025 kHz, 7.177 kHz and 3.0499 kHz provide the absolute widths (relative widths) of 12.899 kHz (0.8429), 5.198 kHz (0.7243) and 1.7846 kHz (0.5851), respectively. In fact, the optimization objective function in Eq. (1a) can successfully drive the evolution to generate the sufficiently wide negative range over *f*_max_.

Interestingly, the three optimized microstructures in Fig. 1(d) exhibit the common geometrical features: (i) multiple solid blocks interconnected by the narrow solid connections, (ii) two centered blocks placed in the *y*-direction, and (iii) several slender rods located along the *x*- or *y*-direction, acting as either the horizontal or the vertical connections. Intuitively, these features are responsible for the strong anisotropy of the dynamic wave responses with respect to the two principal directions. Generally speaking, increasing the thickness of the connections gives rise to the increase of the operating frequency range. On the other hand, the anisotropy degree of the effective dynamic behaviors mainly depends on the symmetries and topologies of the multiple blocks.

### Negative properties and mechanism analysis

**Figure 2:**
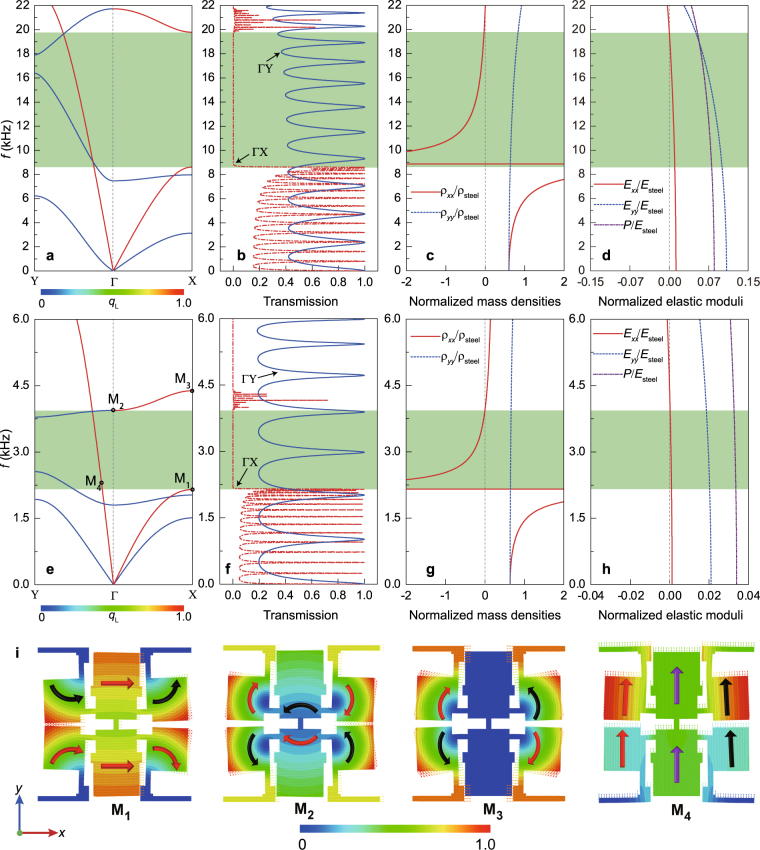
Characterizations of the HEMMs H1 (**a**–**d**) and H3 (**e**–**h**) in Fig. 1(d). (**a**,**e**) Band structures along the ГX- and ГY-directions for the in-plane waves. (**b**,**f**) Transmission coefficients along the two principal directions of a finite HEMM sample for the longitudinal input excitation. (**c**,**g**) Effective mass densities along the *x*- and *y*-directions. (**d**,**h**) Effective elastic moduli. Here, we use ${{q}}_{{\rm{L}}}=|\mathop{{\rm{\Sigma }}}\limits_{{\rm{unitcell}}}{{u}}_{{\rm{L}}}|/\sqrt{{(\sum _{{\rm{unitcell}}}{{u}}_{{\rm{L}}})}^{2}+{(\sum _{{\rm{unitcell}}}{{u}}_{{\rm{T}}})}^{2}}$ to characterize the wave motion for the existing propagating modes. The longitudinal (transverse) wave motions of the eigenstates along the two principal directions can be characterized by the quantity *q*_L_ = 1.0 (*q*_L_ = 0) in Fig. 2(a,e). (**i**) Eigenstates marked in Fig. 2(e) for H3. The eigenstates **M**_**1**_ (*f* = 2151.75 Hz), **M**_**2**_ (*f* = 3939.54 Hz) and **M**_**3**_ (*f* = 4385.25 Hz) correspond to the multipolar and quadrupolar resonances. Here, the colored arrows indicate the corresponding vibration directions of the solid blocks. Since the unit-cell’s boundaries for **M**_**1**_-**M**_**3**_ show the longitudinal vibrations along the *x*-direction, the effective motions are equivalent to the longitudinal waves along the *x*-direction. Unlikely, the eigenstate **M**_**4**_ (*f* = 2260.6 Hz) presents the translations of all blocks, showing the propagation of the longitudinal waves in the *y*-direction.

To demonstrate the negative properties of the optimized HEMMs, we numerically compute the dispersion relations and the wave transmissions, and extract the effective material parameters for H1 and H3, as respectively illustrated in Fig. 2. The method for the determination of the effective material parameters, the wave transmission along the two principal directions, the transmission of the propagating and evanescent elastic waves, and the hyperbolic properties of H2 are presented in the Supplemental Material^38^ for the sake of brevity. In Fig. 2(a,e), the wide directional bandgaps in the ГX-direction occur between the two longitudinal wave bands, while a relatively straight longitudinal wave band is maintained within the same range along the ГY-direction. The negative *ρ*_*xx*_ with the positive *E*_*xx*_ (Fig. 2(c,d,g,h)) can accurately capture the occurrence of the bandgap along the ΓX-direction. The positive *ρ*_*yy*_ with the positive *E*_*xx*_ (Fig. 2(c,d,g,h)) also predicts the existence of the longitudinal wave mode in the ΓY-direction. The transmission properties (Fig. 2(b,f)) along the two principal directions also show that the longitudinal waves cannot propagate within the metamaterial in the ΓX-direction but can propagate along the ΓY-direction. Therefore, the hyperbolic dispersion is resulted from the different characteristics along the two orthogonal principal directions of the HEMM microstructure. For H3 with the bandgap in the ultra-low frequency region when *ρ*_*yy*_ keeps nearly a constant value, *ρ*_*xx*_ turns to be negative within the range of (2.157 kHz, 3.943 kHz). Unlikely, *E*_*xx*_ is always positive in the same range. The HEMM H3 has the simultaneously positive *ρ*_*xx*_ and *E*_*xx*_ in the range of (3.94 kHz, 4.385 kHz), which characterizes accurately the wave modes of the forth band in the ГX-direction. Here, we apply the effective longitudinal modulus *P* = *K* + *μ* (where *K* is the effective bulk modulus and *μ* is the effective shear modulus)^38^ to characterize the effective material behaviors concerning the longitudinal wave motion. Unlike *E*_*xx*_ and *E*_*yy*_, no large decrease of *P* is observed. This, in turn, explains the strong anisotropy of the elastic moduli.

To reveal the physical mechanisms of the negative effective material properties, we investigate the representative eigenstates M_1_-M_4_ marked in Fig. 2(e), see Fig. 2(i). The eigenstate M_1_ in the lower edge of the bandgap has the energy mostly concentrated in the six solid blocks, while the eigenstate M_2_ in the upper edge shows the opposite vibrations. Therefore, the origin of the bandgap in Fig. 2(e) is the result of the enhanced multipolar resonances which generate the negative *ρ*_*xx*_ within the range of (2.157 kHz, 3.943 kHz). The eigenstate M_3_ shows the rotations of the four smaller blocks with the bigger two almost unmoving. This is the typical quadrupolar resonance generating the negative *E*_*xx*_ above 4.385 kHz as shown in Fig. 2(h). As for the ГY-direction, we also display the eigenstate M_4_ of the longitudinal wave band within the range of (2.157 kHz, 3.943 kHz). It is shown here that the *y*-polarized translation dominates the total motion of the unit-cell. From these analyses, we can conclude that the optimized HEMMs can readily control the *x*- and *y*-polarized wave motions independently through the multipolar resonances.

### Subwavelength imaging

**Figure 3:**
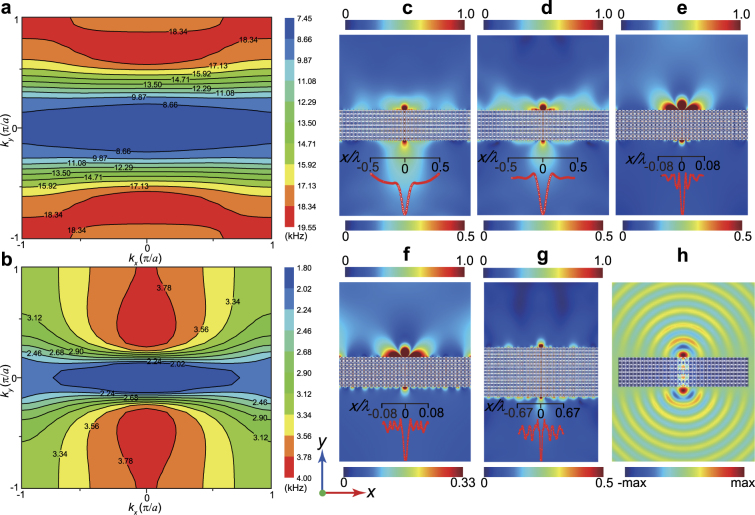
EFCs and imaging results based on the optimized HEMMs. (**a**)–(**b**) EFCs of the third band for H1 and H3 in Fig. 1(d). (**c**)–(**f**) Field magnitude patterns of the longitudinal wave component showing the imaging for the 35 × 8 slabs based on H1 (**c**,**d**) and H3 (**e**,**f**) at the frequencies of (13 kHz, 14 kHz) and (2.3 kHz, 3.1 kHz). (**g**) Field magnitude patterns of the longitudinal wave component showing the imaging for the 35 × 15 slab based on H1 at 12.96 kHz. (**h**) The acoustic pressure field for the imaging of a 35 × 8 slab based on the HEMM H1 with the water as the background material at 13 kHz. The imaging resolutions in (**c**)–(**h**) are FWHM = 0.178*λ*, 0.167*λ*, 0.0156*λ*, 0.0253*λ* and 0.074*λ* and 0.34*λ*, respectively. The point wave source is located in the position 0.02 m away from the upper side of the HEMM slab. The normalized intensity profiles of the images are displayed in the subgraphs (**c**)–(**g**).

To demonstrate the hyperbolic dispersion, we illustrate the EFCs of the HEMMs H1 and H3 in Fig. 3(a,b), respectively, leaving details of H2 to the Supplemental Material^38^. Due to the strong anisotropy, the EFCs show a distinctive hyperbolic shape. When the incident waves launch into the HEMMs, the refracted group velocity, which is perpendicular to the contours and pointing away from the interface, must be in a negative direction^9^. That is, the negative refraction for the propagating wave mode appears at the interface between the HEMM and the background material (stainless steel). It is observed from Fig. 3(a,b) that the curvature of the curve becomes larger with the frequency increasing. Particularly, the bottoms of the hyperboloids clearly show the extremely flat profiles in a broadband frequency range, which can contribute to the energy funneling phenomenon^3,4^ with a large bandwidth. The flatter curves over the whole wave vector range give rise to the larger group velocities. Furthermore, three optimized HEMMs (H1-H3) possess the broadband hyperbolic dispersions with the bandwidths of 10.651 kHz (H1), 4.095 kHz (H2) and 1.755 kHz (H3), respectively. Independent of the subwavelength or deep-subwavelength scales, all these values outperform the previously reported frequency bandwidths of the HEMMs^10,11^. Certainly, we can freely scale up or down the optimized microstructures for the operation at much lower or higher frequencies. Since our present study only focuses on the longitudinal wave propagation, the evident hyperbolic dispersions shown in Fig. 3(a,b) validate that our proposed topology optimization strategy is robust for the longitudinal waves, no matter whether the transverse waves exist or not.

To validate the imaging performance of the optimized HEMMs, we demonstrate the typical hyperlensing effect of the longitudinal waves at various operating frequencies in Fig. 3(c–f). Here, we investigate the optimized HEMMs H1 (Fig. 3(c,d)) and H3 (Fig. 3(e,f)). A HEMM slab with 35 × 8 unit-cells surrounded by the background material (stainless steel) is considered. In all considered cases, a point source of the longitudinal wave is applied on the position 0.02 m away from the upper side of the HEMM slab. We consider the operating frequencies of 13 kHz (Fig. 3(c)) and 14 kHz (Fig. 3(d)) for H1, as well as 2.3 kHz (Fig. 3(e)) and 3.1 kHz (Fig. 3(f)) for H3. The obvious difference between the sizes of the source and the image as shown in the figures is due to the inevitable incident wave reflection which is caused by the impedance mismatch between the background material (stainless steel) and the metamaterials. However, from the intensity profiles of the images (the red curves in Fig. 3c–g), we can observe clearly that the wave propagates through the HEMM and yields an image of the source on the other side of the slab. We obtain the full width at the half maximum (FWHM) of the four images as 0.178*λ* (Fig. 3(c)), 0.167λ (Fig. 3(d)), 0.0156λ (Fig. 3(e)) and 0.0253λ (Fig. 3(f)). Moreover, the lower the operating frequency is, the higher imaging resolution the optimized HEMM can realize. Surprisingly, all these imaging resolutions, which are much higher than the diffraction limit, exceed the previously reported values of the HEMMs proposed by Oh *et al*.^8^, Zhu *et al*.^10^ and Lee *et al*.^11^. The reported super-high resolution of 0.0156*λ* (~*λ*/64) represents the record even in the field of EMMs. We believe that these hyperlensing properties are realized owing to the hyperbolic dispersions with the extremely anisotropic mass densities.

Moreover, based on the optimized microstructures, we can adjust the thickness of the lens to meet the Fabry-Pérot resonant condition^39^ for standing wave excitation. Figure 3(g) displays the imaging simulation for a 15 × 35 EMM slab based on the HEMM H1 at 12.96 kHz. Clearly, the considered novel hyperlens gives the enhanced imaging transmission. More importantly, the obtained imaging resolution (FWHM = 0.074*λ*) is much higher than that (FWHM = 0.178*λ*) in Fig. 3(c). This improvement results from more standing waves excited in the lens. In fact, it is in principle possible to increase the thickness of the lens in the optimized HEMM H3 for a higher resolution smaller than 0.0156*λ*.

It is noticed here that the transmission in the above HEMM is very low and therefore the image is very weak due to the large impendence mismatch. This is because that the impedance match condition is not included in the optimization approach. If this condition is considered, a metamaterial with a higher transmission performance may be obtained. A typical example is displayed in Sec. 6.3 of the Supplemental Material^38^. It is seen that the transmission is obviously improved. However, a better impedance match often requires the simultaneously large mass density and stiffness, which is rather difficult to realize within the ultra-low frequency region (*λ* > = 90*a*).

We further mention that an imaging of the designed HEMM with a high resolution can be also realized in a fluid medium. Figure 3(h) presents the imaging of the hyperlens based on HEMM H1 with the water instead of the stainless steel as the background material. The imaging resolution with FWHM = 0.34*λ* is lower than that (FWHM = 0.178*λ*) in Fig. 3(c). However, a nearly perfect imaging transmission is achieved with only a small amount of the wave energy being reflected because of the ideal impedance matching.

**Figure 4:**
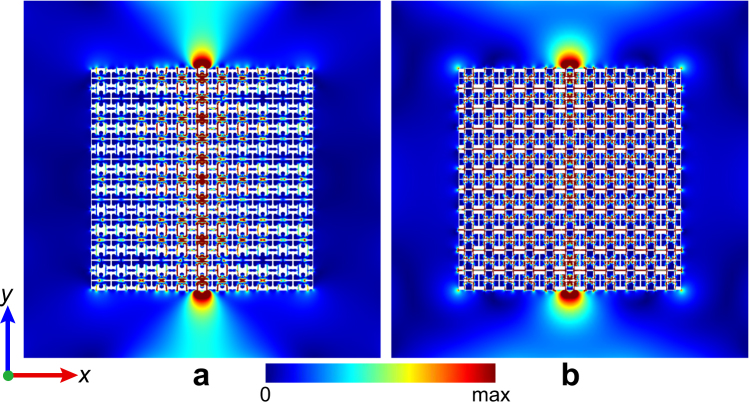
Wave propagations in the HEMMs. Field magnitude patterns of the longitudinal wave component propagating in H1 (**a**) and H3 (**b**) at 13 kHz and 2.3 kHz. The imaging resolutions in the upper and lower boundary areas in (**a**) and (**b**) are FWHM = 0.169*λ* and 0.02*λ*, respectively. A point source of the longitudinal wave is applied in the center of the 11 × 11 HEMM slab. Two images occur at the upper and lower sides of the slab. However, no visible waves are found at the left and right boundaries.

Since the hyperbolic dispersion is responsible for the above hyperlensing effect, we present the longitudinal waves propagating in the optimized HEMMs H1 and H3 at 13 kHz and 2.3 kHz, respectively, to verify the strongly anisotropic wave motions, see Fig. 4. For this purpose, we apply a point source of the longitudinal wave in the center of a 11 × 11 HEMM slab. Here, it can be clearly recognized that the longitudinal wave propagates only along the *y*-direction, which coincides with the hyperbolic EFCs. It is very interesting to note that the images in these two cases occur nearly in the same regions, indicating the fact that the optimized HEMMs have a stable focused energy at those frequencies with very flat EFCs, and all the widths of the four energy concentration areas are about 1.5*a*. Presumably, this is due to the similar boundary structures which can transmit the similar wave motions at the interfaces between the metamaterial and the background material. However, from the viewpoint of the topology optimization, it is unlikely to further change the topologies of the boundaries in the optimized metamaterials. Therefore, the similar capacities of the wave focusing in Fig. 4 imply that the three HEMMs in Fig. 1(d) may have the similar ultra-high resolution imaging abilities at the corresponding wavelength scales. We stress here that the resolution of *λ*/64 shown in Fig. 3(e) possibly represents the highest hyperlensing performance approaching to the limit within the proposed topology optimization framework.

**Figure 5:**
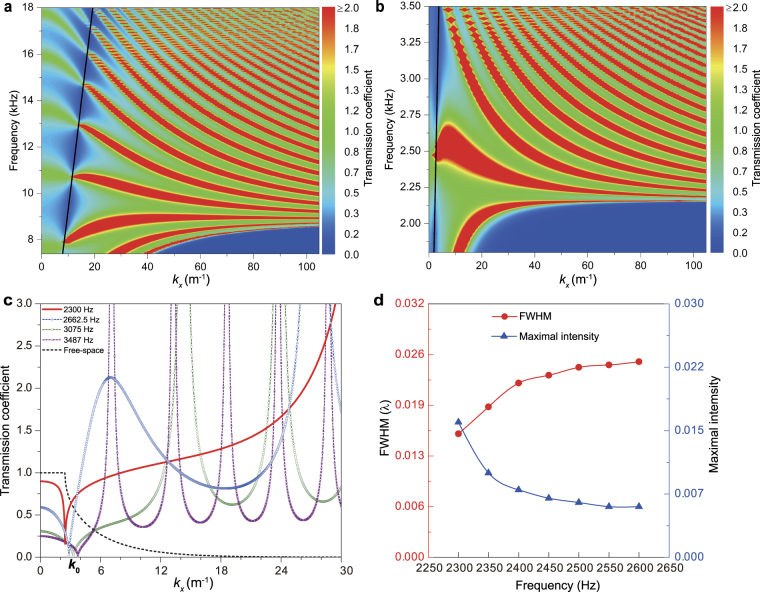
Enhancement of the evanescent waves and imaging features. (**a**)–(**b**) Frequency and wave-vector dependence of the transmission through a layer (thickness 8*a*) of the HEMM H1 (**a**) and H3 (**b**) for both propagating and evanescent waves. The two skew lines in (**a**) and (**b**) represent the dispersion curves (*ω* = *c*_L_×*k*_0_, *c*_L_ is the longitudinal wave velocity of the stainless steel) for the longitudinal waves in the background material. (**c**) Transmission coefficients for H3 at several frequencies and for the free-space at 2300 Hz. (**d**) Performance of the imaging resolution and maximal intensity on the focal plane based on H3 slab consisting of 35 × 8 unit-cells at different operating frequencies. Here, the propagation constant of the fundamental waveguide mode is defined as *k*_0_ = 2π/*λ*^39^. If *k*_*x*_ ≤ *k*_0_, the transmission coefficient characterizes the transmission property for the propagating waves, while for *k*_*x*_ > *k*_0_ the corresponding waves represent the evanescent waves^9^. For comparison, the result for the free-space (stainless steel) at 2300 Hz is also presented in (**c**). It is easy to distinguish the evanescent wave transmission with a fast attenuation in the free-space from the propagating wave transmission.

To reveal the reason for the super-resolution imaging, we consider the zero-order transmission coefficient *T* (more details on *T* are given in the Supplemental Material^38^) of a plane elastic wave and qualitatively evaluate the transmission of both propagating and evanescent waves^38^ through a metamaterial layer of the optimized HEMMs embedded into a background material (so-called free-space) The metamaterial layer has a thickness of 8*a* and the background material is the stainless steel. Figure 5(a,b) illustrate the transmission coefficients of the HEMMs layers H1 and H3 over certain frequency ranges versus the wave component *k*_*x*_. According to the definition of *T*^38^, a value of *T* larger than 1.0 means that the propagating or evanescent waves are enhanced. From Fig. 5(a), it is clearly seen that the transmission coefficient is large enough (*T* > 1.0) for a large *k*_*x*_ in the negative *ρ*_*xx*_ range. However, in the high-frequency region, a large transmission coefficient cannot occur in a wide range of the wave vector, and the transmission coefficient decreases gradually as the frequency rises. The similar behavior is observed in Fig. 5(b). At a certain operating frequency below the negative *ρ*_*xx*_ range, the transmission coefficient can be large only in a narrow wave vector region, especially when the frequency approaches 2.1576 kHz. In particular, the transmission coefficient shows a pronounced increase around 2.5 kHz in a wide *k*_*x*_ range. Therefore, the results in Fig. 5(a,b) demonstrate that the evanescent waves are enhanced significantly in the HEMMs H1 and H3. The flat hyperbolic dispersions in Fig. 3(a,b) ensure the enhancement of the evanescent waves over a wide range with a large *k*_*x*_^7^. In the process of the wave focusing, the optimized HEMMs with the hyperbolic dispersions will convert the evanescent wave components containing the subwavelength information into the propagating wave components and transfer the energy to the focal plane of the image^7,9,40^. For the abovementioned reasons, it can be concluded that it is the extreme enhancement of the evanescent waves which results in the super-high imaging resolution of *λ*/64.

To explicitly reveal the effect of the frequency on the transmission, we show the transmission coefficients in the HEMM H3 at four frequencies 2300 Hz, 2662.5 Hz, 3075 Hz and 3487 Hz in Fig. 5(c). The corresponding effective mass densities in the *x*-direction are *ρ*_*xx*_ = −24526 kg m^−3^, −5303 kg m^−3^, −1948 kg m^−3^ and −698 kg m^−3^, respectively. Figure 5(c) reveals that, because of the imperfect effective impedance matching, the optimized metamaterials in the four considered cases have lower transmission coefficients for the propagating waves than the reference result. However, the strongly enhanced transmission of the evanescent waves is excited simultaneously in these four cases. This implies that, contrary to the free-space (stainless steel) case, the optimized HEMM H3 has the competence of generating a high imaging resolution. Moreover, as the frequency increases, the transmission of the propagating waves gradually decreases resulting from the worse effective impedance matching. The enhancement extent of the evanescent waves reduces synchronously because the smallest transmission coefficient in the evanescent region decreases obviously. The case at 2300 Hz distinctively shows the best and extreme enhancement property over the considered wave vector range. So, the simultaneously best imaging transmission and resolution of the HEMM H3 generally exist at lower frequencies within the hyperbolic frequency range. The other two optimized HEMMs H1 and H2 also have a similar feature. In order to examine the effect of the transmission of the propagating and evanescent waves on the imaging, we show the performance of the imaging resolution and the maximal intensity on the focal plane based on the constructed HEMM H3 slab consisting of 35 × 8 unit-cells at different operating frequencies in Fig. 5(d). Interestingly, the resolution keeps a high level as the frequency goes up, although a certain variation exists. In the narrow frequency range (2300–2600 Hz), the resolution and the imaging transmission (maximal intensity) present a trade-off trend. Moreover, the stability of the resolution and imaging transmission indicates the strong robustness of the optimized HEMMs. Since the resonance can enhance the evanescent waves in the hyperlens^40^, we presume that the abovementioned extreme enhancement of the evanescent waves originates physically from the multipolar resonances which can essentially make the *x*- and *y*-polarized vibrations coupled together.

## Conclusions

Single-phase metallic metamaterials with broadband hyperbolic dispersions for longitudinal waves are presented in this paper. Based on the effective mass density, a topology optimization strategy is developed for the design of the metamaterials. It is shown that the special multipolar resonances can guarantee the occurrence of the strongly anisotropic effective mass density with negative values along one direction in the deep-subwavelength frequency region (*λ*/*a* ≈ 10~90). The representative structural topology provides guidance for the engineering of HEMMs and even more metamaterial devices with complex functionalities. All the imaging simulations of the longitudinal waves in the optimized hyperlens demonstrate their transfer ability for the subwavelength information, which results in the extremely high resolutions beyond the diffraction limit. Moreover, benefited from the extreme enhancement of the evanescent wave transmission, a super-high imaging resolution of about *λ*/64 is realized based on the optimized HEMMs at the ultra-low frequency level. In addition, topology optimization offers an explicit choice to controlling the relative impedance of microstructure for enhancing imaging transmission. Owing to the special exploration ability of our design strategy based on the topology optimization, the optimized HEMMs in this paper exhibit several novel undiscovered structural topologies, the record broadband frequency ranges and the record imaging resolutions in the field of EMMs, to the best knowledge of the authors. Thus, the present HEMMs may open a new way for high-performance metamaterials in many potential innovative applications such as medical imaging, sensing and nondestructive testing. The proposed design strategy can be easily extended to design hyperbolic metamaterials and even metamaterials with the arbitrary effective parameters for other wave counterparts.

## Methods

### Genetic algorithm (GA) for constrained optimization

The improved single-objective genetic algorithm (GA)^33^ is adopted to solve the optimization problem described by Eqs (1a)-(1h). Each binary chromosome involved in the GA corresponds to a microstructure formed by a coarse grid with 30 × 30 pixels (square finite elements regarding the material phase 0 or 1). The search space for the optimization has 2^*N* × *N*^ design variables. With the orthotropic symmetry, the total number of the possible structures is reduced to 2^*N* × *N*/4^. In the GA procedure, a random initial population containing *N*_p_ = 30 individuals (chromosomes) is created. The “abuttal entropy filter” for filling up some isolated voids and removing some isolated elements is applied to improve the structural strength. Then, the objective function value *SN* is computed for each individual. The constrained optimization is formed after considering all constraint-violating cases. If the *i*th individual is a feasible solution, then the final fitness evaluation which is equal to *SN* is defined as

2a $$fitness=S{N}_{i}$$

Otherwise, if the individual cannot meet some constraints, then the fitness is determined by

2b $$fitness=\,{\rm{\min }}(S{N}_{1},\,S{N}_{2},\cdots S{N}_{NP})-\sum _{j=1,2\cdots S}|c{v}_{j}|,$$

where *S* is the number of the violated constraints; *cv* represents the violation extent for a certain constraint. The algorithm gradually employs several genetic operations, including the reproduction for the tournament selection with the size of the competition group *N*_ts_ (=18), the crossover with the crossover probability *P*_c_ (=0.9) and the mutation with the mutate probability *P*_m_ (=0.03/0.005 for the coarse/fine grid) to generate the offspring population. The elitism strategy^33^, which preserves the best individual in the current generation as an elitism and replaces the worst one in the next generation by the elitism, is utilized to accelerate the optimization. The optimization process is repeated until a prescribed large number (e.g. 1000) of generations is finished. Finally, the GA produces an optimized microstructure which can be regarded as a “seed” individual for the new round of the optimization in a finer grid with 60 × 60 pixels for the better description of the structural boundaries. After the iterations, the final optimized microstructure is generated.

### Numerical simulations

The simulations of the dispersion relations, wave transmission, effective material parameters and eigenstates were performed by the commercial finite element software ABAQUS 6.11–1. The simulations of the wave imaging and propagation through the hyperlens based on the optimized HEMMs were conducted by COMSOL Multiphysics 4.4. The optimization procedures were implemented on a Linux cluster with Intel Xeon X5650 Core @ 2.66 GHz.

## Electronic supplementary material

Supplemental Material
