# Supplementary material for: Broadband single-phase hyperbolic elastic metamaterials for super-resolution imaging
Source: Sci Rep. 2018 Feb 2;8:2247. doi: 10.1038/s41598-018-20579-8 (PMC5797129; doi:10.1038/s41598-018-20579-8)
Supplement: Supplementary file 1 — Supplemental Material [file 41598_2018_20579_MOESM1_ESM.pdf]

Broadband single-phase hyperbolic elastic metamaterials for super-resolution imaging

Hao-Wen Dong[1,2], Sheng-Dong Zhao[2], Yue-Sheng Wang[2,*], Chuanzeng Zhang[3,*]

[1]Department of Applied Mechanics, University of Science and Technology Beijing, Beijing 100083, China
[2]Institute of Engineering Mechanics, Beijing Jiaotong University, Beijing 100044, China
[3]Department of Civil Engineering, University of Siegen, D-57068 Siegen, Germany

Supplemental Information

1. Determination of the effective material parameters

The effective medium theory [1] is adopted to describe the dynamic behaviors of the elastic metamaterials (EMMs) [1, 2]. Since topology optimization involves extremely complex geometries, a numerical determination [1] of the effective material parameters is often applied. Under the long-wavelength assumption [1, 2], we evaluate the effective material parameters of the metamaterials by applying a global displacement field on the unit-cell boundaries with the displacement phase difference being ignored.

According to the Newton's second law, the mass density tensor can be determined by [1, 2]

$$\begin{bmatrix} F_x^0 \\ F_y^0 \end{bmatrix} = -\omega^2 V \begin{bmatrix} \rho_{xx} & 0 \\ 0 & \rho_{yy} \end{bmatrix} \begin{bmatrix} U_x^0 \\ U_y^0 \end{bmatrix}, \qquad \text{(SQ1)}$$

where F_x^0 and F_y^0 represent the total induced forces on the boundaries along the x- and y-directions, respectively; ω is the angular frequency; V denotes the volume of the effective medium; ρ_{xx} and ρ_{yy} are the effective mass densities in the x- and y-directions, respectively; and the applied displacement field is represented by U_x^0 and U_y^0. The off-diagonal elements of the mass density tensor are zero because of the orthogonal symmetry of the unit-cell's microstructure.

By applying the corresponding unit eigenstates, the effective stiffness E_{xx} can be determined from the energy equivalence between the unit-cell and the effective medium by

$$\Sigma(F_x^* U_x^*) = E_{xx} V , \qquad \text{(SQ2)}$$

where F_x^* and U_x^* are the induced nodal force and displacement along the x-direction on the unit-cell boundaries under the applied time-harmonic displacement field of $U_x^* = xe^{iwt}$ on the left and right boundaries and $U_y^* = 0$ on the upper and lower boundaries. The determination of E_{xy} and E_{yy} can be performed in the similar way. It

* Corresponding author. Tel.: +86 10 51688417; fax: +86 10 51682094. *E-mail address:* yswang@bjtu.edu.cn (Y. S. Wang).
* Corresponding author. Tel.: +49 271 7402173; fax: +49 271 7404074. *E-mail address:* c.zhang@uni-siegen.de (Ch. Zhang).

is noted here that we focus on the non-propagating waves along one principal direction to obtain the hyperbolic dispersion. Therefore, the effective elastic modulus E_{xx} or E_{yy} should be completely generated by the induced normal motion along the x- or y-direction. In this case, the applied eigenstates are different from the work by Liu et al. [1]. However, we can also utilize the longitudinal wave modulus $P=K+\mu$ (where K is the effective bulk modulus and μ is the effective shear modulus) to characterize the whole effective behaviors concerning the longitudinal wave motion. The detailed process for the numerical determination of K and μ can be found in the work by Liu et al. [1].

2. Wave transmission along the two principal directions

Figure S1 shows the wave transmission computation of an incident elastic wave in an optimized metamaterial. Let us consider a plane time-harmonic longitudinal wave along the y-direction $u_y^{in} = u_y e^{i(k_0 y - \omega t)}$ which is incident from the left into the elastic metamaterial, where u_y is the amplitude of the incident wave field, k_0 represents the wave vector in the background material (stainless steel), and ω is the operating angular frequency [11]. For the sake of simplicity, the time-harmonic factor $e^{-i\omega t}$ is omitted in the following. Then, the displacement field in the left background region is determined by $u_y^r = u_y^{in} + R u_y e^{-ik_0 y}$, while in the right background domain the corresponding displacement field is given by $u_y^t = T u_y e^{ik_0 (y-d)}$ where R and T are the reflection and transmission coefficients, respectively [11]. The similar computational procedure can be used for the case of an incident wave along the x-direction.

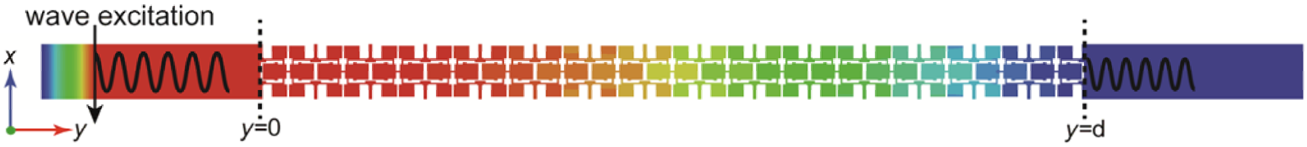

Figure S1 | Transmission computation of an incident elastic wave. The optimized metamaterial is located in the middle region (y=0-d) which is surrounded by the background material. The whole domain is terminated with the infinite elements, and the periodic boundary conditions are assumed on the upper and lower boundaries.

3. Transmission of the propagating and evanescent elastic waves

All our optimized metamaterials show an outstanding deep-subwavelength imaging benefited from the transmission enhancement of the evanescent waves, which can convert the subwavelength information into the propagating components. In order to characterize the wave transmission [3, 4], we perform the numerical analysis of the propagating and evanescent waves transmitting through a layer of the anisotropic effective medium between two semi-infinite homogeneous stainless spaces (free-space). In this paper, we only focus our attention on the hyperbolic dispersion of the longitudinal waves by neglecting the transverse waves. Due to the nearly constant value, the longitudinal wave modulus P can be utilized to describe the whole longitudinal wave motion. In this case, the anisotropic mass density dominates the formation of the hyperbolic dispersion of the elastic waves. Referring to the study reported by Zhu et al. [5], the dispersion relation for the longitudinal wave propagating in the EMMs with an anisotropic mass density can be expressed by

$$\frac{k_x^2}{\rho_{xx}}+\frac{k_y^2}{\rho_{yy}}=\frac{\omega^2}{P}, \tag{SQ3}$$

where k_x and k_y are the components of the wave vector in the anisotropic effective elastic medium along the x- and y-directions, respectively. The transmission coefficient T of the propagating and evanescent waves is determined as [3, 4]

$$T=\left|\frac{4Z_y Z_{0y} e^{ik_y L}}{(Z_y+Z_{0y})^2-(Z_y-Z_{0y})^2 e^{2ik_y L}}\right|, \tag{SQ4}$$

where $Z_y=\omega\rho_{yy}/k_y$ and $Z_{0y}=\omega\rho_0/k_{0y}$ are the wave impedances; ρ_0 is the mass density of the background medium; L is the thickness of the layer in the optimized metamaterial; $k_{0y}=\sqrt{k_0^2-k_x^2}$ is the component of the wave vector in the free-space; and $k_0=2\pi/\lambda$ denotes the propagation constant of the fundamental waveguide mode [6]. If $k_x\leq k_0$, the transmission coefficient T characterizes the transmission property of the propagating waves, while for $k_x>k_0$ the corresponding waves represent the evanescent waves. For a microstructure generated from the GA, substituting the effective wave impedances into Eq. (SQ4) yields the transmission coefficient T at a fixed frequency.

4. Description of the objective function

Inspired by the mechanism that the dipolar resonances [1, 2, 5, 7] can produce a negative mass density, we should search for the topology of the microstructure to induce a resonance at a certain frequency. The effective mass density ρ_{xx} increases to the positive infinity when the operating frequency below the resonant frequency rises. However, a negative ρ_{xx} occurs when the operating frequency above the resonant frequency increases. As a result, a negative ρ_{xx} in a certain frequency range is obtained by the topology optimization scheme [8, 9, 10]. To achieve a wider negative range in the lower-frequency region, it is necessary to push down the resonant frequency and make the overall positive ρ_{xx} smaller [7]. If we compute the effective parameters at the sampling frequencies which are equably distributed in the target operating frequency range ($f_{\min}$, $f_{\max}$), then the most important driving force to generate a negative ρ_{xx} in a wide frequency range is to enlarge the gap between the maximal and the minimal positive values at these sampling frequencies. Therefore, $-\min(\rho_{xx}^{m+})/\max(\rho_{xx}^{m+})$ will be maximized to achieve this purpose. Once the resonances take places within the target frequency range, the values of ρ_{xx} at several sampling frequencies become negative. In this case, N is introduced in Eq. (1a) to ensure the individual with a negative ρ_{xx} to be completely superior to others, thus guiding the evolution to find out more individuals with larger N. In this way, topology optimization can explore metamaterials with a broadband negative mass density.

5. Description of the constraints

In our optimization formulation for the negative ρ_{xx}, we introduce seven constraints (1b)-(1h). The corresponding physical reasons for these constraints are given as follows.

- **Constraints (1b)-(1d):** Warranty for the longitudinal wave motion in the y-direction

To realize the single-negativity for the hyperbolic dispersion, we have to guarantee the eigenstate motions for the positive ρ_{yy}, E_{xx} and E_{yy} simultaneously when hunting for the negative ρ_{xx}. In addition, E_{yy} should be larger than E_{xx} to generate the longitudinal band along the y-direction over a large frequency range in which a bandgap

in the x-direction is opened. These factors will be taken into account by the constraints (1b)-(1d), which can ensure the emergence of the longitudinal wave motion along the y-direction.

- **Constraints (1e):** Warranty for the exclusive longitudinal wave motion in the y-direction

When ρ_{xx} turns negative from positive, both E_{11} and E_{22} usually decrease, owing to the increase of the void fraction in the metamaterial. However, the multipolar resonances revealed in this paper can effectively induce not only a negative ρ_{xx} but also a negative E_{xx} (although very small). If E_{xx} is positive and close to zero, however, it means that the vibration in the x-direction can be easily coupled with the excited longitudinal wave motion along the y-direction. This results in the complex hybrid vibration modes containing the longitudinal and transverse wave motions. That is, the longitudinal band along the y-direction may show the quasi-longitudinal wave motion. Therefore, we introduce the constraint (1e) to control the coupling stiffness E_{12} in order to obtain an exclusive longitudinal wave motion in the y-direction.

- **Constraint (1f):** Warranty for a purely translational motion in the y-direction

Normally, we can find the strong "local rotation" which shows the off-diagonal feature of the effective mass density and stiffness tensors beyond the classical linear elasticity theory. In particular, the topology optimization usually involves many extremely complex microstructures which can bring more serious "local rotation" problems. Accordingly, we have to introduce the constraint (1f) to make sure that the induced behavior is completely the translation motion in the y-direction when retrieving ρ_{yy}. Otherwise, the performance of ρ_{yy} cannot be coincident with the dispersion relation.

- **Constraint (1g):** Warranty for a strong anisotropy

A strong anisotropy is the most important feature of the hyperbolic metamaterials. To obtain the strongly anisotropic mass densities along the two principal directions, with ρ_{yy} as a nearly constant value when ρ_{xx} varies from the positive infinity to the negative infinity. Because a nearly constant ρ_{yy} indicates a nearly non-dispersive performance along the y-direction, the extremely different features in the two directions naturally contribute to the hyperbolic dispersion with a maximal anisotropy. So, the constraint (1g) is introduced here to achieve this goal. The numerical tests show that δ_ρ=1.37 can effectively balance the requirements of the strong anisotropy and the large feasible solution space.

- **Constraint (1h):** Warranty for a sufficient stiffness

The constraint (1h) is introduced to design only such a metamaterial with a sufficient stiffness for practical manufacturing. Naturally, this constraint can also suppress the mesh-dependency problem [8, 10] encountered in topology optimization.

6. Complementary results of the optimized HEMMs

6.1. Evolutionary history and convergence of the topology optimization strategy

To check the convergence of the present topology optimization strategy, Figure S2 presents the evolutionary history of the maximal fitness as a function of the generation number for the optimized HEMM H3 in Fig. 1(d).

The metallic structure without any void is taken as the initial "seed" structure, see snapshot of the generation 0 (G=0). From the generation G=0 to G=61, the snapshots show that the GA can quickly capture the beneficial geometry at the early evolution, i.e., thin left and right boundaries, to increase the gap between the minimal and the maximal positive ρ_{xx}. The change from the generation G=61 to G=145 implies that the solid blocks are useful to further enlarge the gap. A significant improvement of *SN* takes place from the generation G=145 (*SN*=−0.7732) to G=209 (*SN*=0.7663). This implies that the geometry of the narrow boundaries with four big solid blocks and two small solid lumps in the center is effective to generate a negative ρ_{xx} at ultra-low frequencies. When the local connections between the blocks and the boundaries get narrower from the generation G=209 (*SN*=0.7663) to G=229 (*SN*=2.6583), the frequency range with a negative ρ_{xx} becomes wider. Then, this increasing tendency can be enhanced by removing more solid parts of the connections from the generation G=229 (*SN*=2.6583) to G=305 (*SN*=3.631). The negative ranges will increase from the generation G=305 (*SN*=3.631) to G=553 (*SN*=4.636) because of the growth of the two central lumps. At this stage, the evolution in the coarse grid begins to converge. Mapping the topology from a coarse grid into a finer one will lower *SN* at some extent. In the fine grid, the GA can commendably make the structural edges clearer and more smooth. From the generation G=1004 (*SN*=4.612) to G=2000 (*SN*=4.7623), the structure turns to possess the extreme narrow connections and sufficiently large solid blocks. Overall, the evolutionary history in Fig. S2 evidently demonstrates the significance of the typical structural features on the generation of a broadband negative ρ_{xx}.

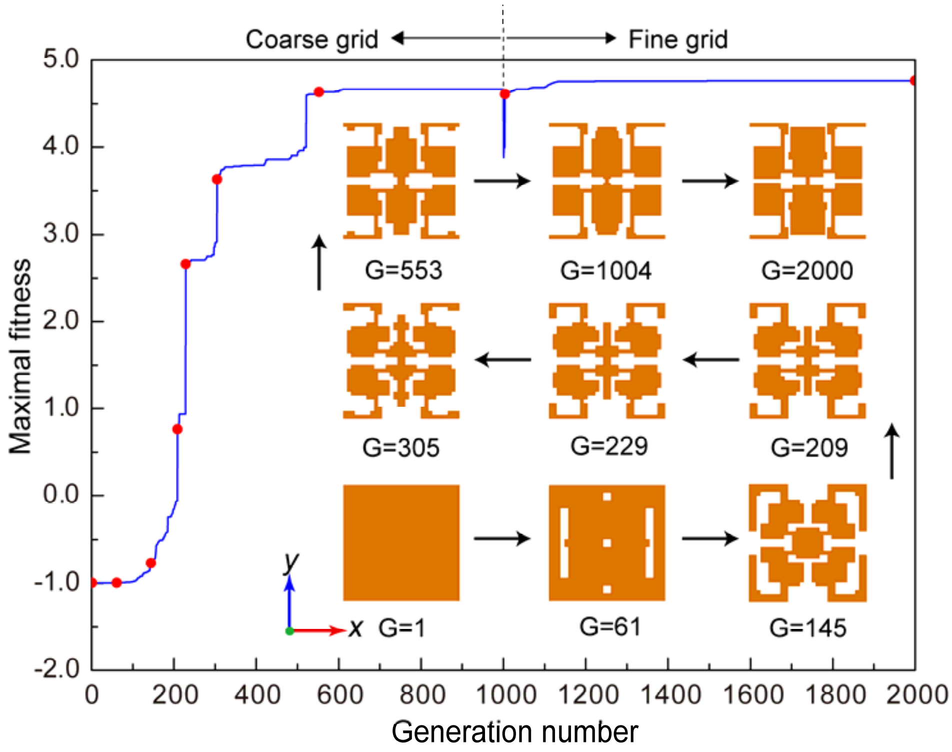

Figure S2 | Evolutionary history for the generation of the HEMM H3 in Fig. 1(d). Snapshots of some representative topologies during the optimization are included. The objective function values (*SN*) of nine microstructures are −0.9988 (G=0), −0.9969 (G=61), −0.7732 (G=145), 0.7663 (G=209), 2.6583 (G=229), 3.631 (G=305), 4.636 (G=553), 4.612 (G=1004) and 4.7623 (G=2000), respectively.

6.2. Hyperbolic properties of the optimized HEMM H2

For completeness to demonstrate the hyperbolic dispersion for all optimized HEMMs in Fig. 1(d), we illustrate in Fig. S3 the corresponding dispersion relations, transmission properties of the longitudinal waves and the effective material parameters for H2. It is clearly seen that the different physical quantities match each other

very well. The negative ρ_{xx} with the positive E_{xx} can accurately capture the occurrence of the bandgap along the ΓX-direction. The positive ρ_{yy} with the positive E_{xx} also predicts the existence of the longitudinal wave mode in the ΓY-direction. The transmission properties along the two principal directions also show that the longitudinal waves cannot propagate within the metamaterial in the ΓX-direction but can propagate along the ΓY-direction. Unlike the results in Fig. 2, the band structure in Fig. S3(a) shows that only the longitudinal wave mode exists along the ΓY-direction in the ΓX-directional bandgap range.

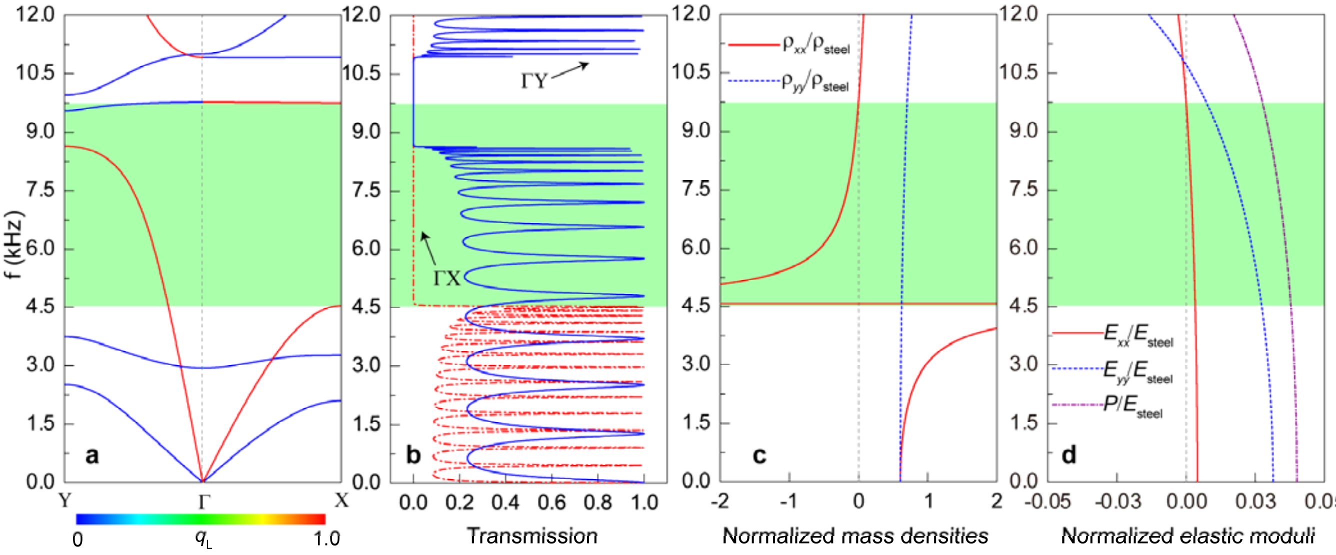

Figure S3 | Characterizations of the HEMM H2 in Fig. 1(d). (**a**) Band structure along the ΓX- and ΓY-directions for the in-plane waves. (**b**) Transmission coefficients along the two principal directions of a finite HEMM sample for the longitudinal input excitation. (**c**) Effective mass density along the *x*- and *y*-directions. (**d**) Effective elastic moduli.

In addition, we illustrate in Fig. S4(a) the EFCs of the third band for H2. We can clearly observe the broadband hyperbolic dispersions. Furthermore, the EFCs become very flat at low frequencies as well. Since we only focus on the longitudinal wave propagation, the optimized metamaterials can act as the HEMMs as long as the EFCs for the longitudinal waves have the hyperbolic shapes. So the evident hyperbolic dispersions shown in Figs. 3(a), 3(b) and S3(a) validate that our proposed optimization formulation described by Eqs. (1a)-(1h) is robust for the longitudinal waves, no matter whether the transverse wave propagation exists or not. To further confirm the hyperbolic properties of H2, Figs. S4(b) and S4(c) show its subwavelength imaging at 5 kHz and 5.5 kHz, respectively. In these two cases, the imaging resolutions are as high as 0.1λ and 0.098λ, respectively. Compared with the imaging resolutions of H1 and H3, we can reasonably conclude that the frequency dominants the ability of the imaging resolutions. Furthermore, the optimized multipolar resonant microstructures in Fig. 1(d) can maintain the excellent hyperlensing effect over the sufficiently wide wavelength range. Figure S4(d) presents the longitudinal wave propagation in H2 at 5 kHz to identify its anisotropic property. It is clear that the longitudinal wave indeed propagates only along the *y*-direction, while no energy can come out from the metamaterials in the *x*-direction. In fact, the hyperbolic curves over large wave vector ranges imply that the metamaterial H2 can realize the negative refraction within the wide angle ranges. Then almost all waves can be highly focused on the top and bottom boundaries. Therefore, like H1 and H3 in Fig. 1(d), the optimized HEMM H2 also shows the broadband hyperbolic dispersion, highly anisotropy and prominent super-resolution imaging. All these benefits show the effectiveness and universality of the topology optimization scheme presented in this paper.

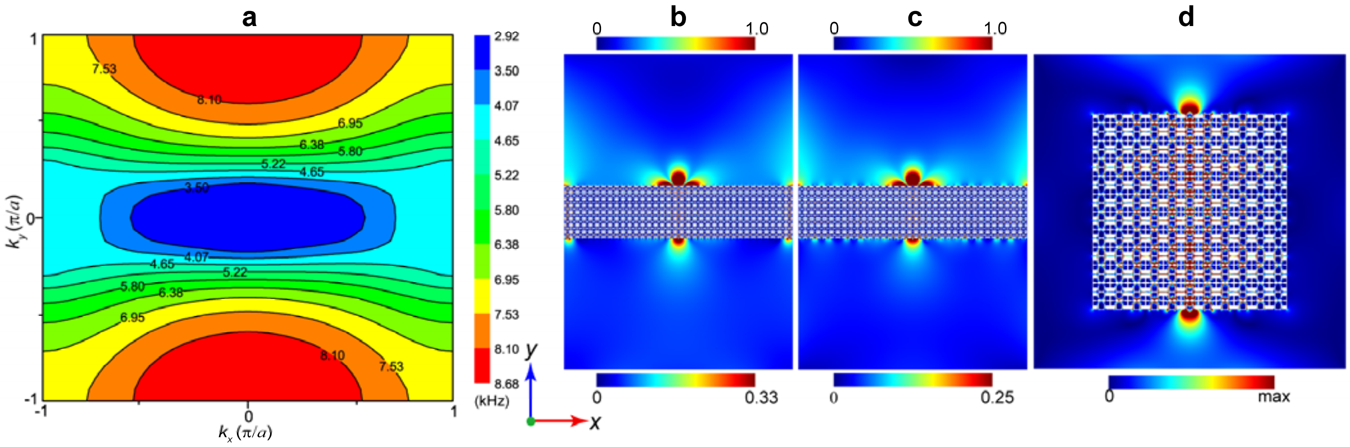

Figure S4 | EFCs and wave propagation based on the HEMM H2. (**a**) EFCs of the third band. (**b**)-(**c**) Field magnitude patterns of the longitudinal wave component showing the imaging for the 35×8 slabs based on H2 at 5 kHz and 5.5 kHz. The imaging resolutions in (b)-(c) are FWHM=0.1λ and 0.098λ, respectively. The point wave source is located in the position 0.02 m away from the top side of the HEMM slab. (**d**) Field magnitude patterns of the longitudinal wave component propagating in H2 at 5 kHz. A point source of the longitudinal wave is applied in the center of the 11×11 HEMM slab. The imaging resolution in the top and bottom boundary areas is FWHM=0.036λ.

6.3. Optimization with impedance match constraint

A high wave transmission is very important for the wave imaging. Generally, in terms of the microstructural design, a better impedance match can improve the imaging transmission regardless of the superlens thickness. When designing the HEMMs, one common approach is to introduce a very simple relative impedance $Z_{\mathrm{R}}=\sqrt{(\rho_{yy}E_{yy})/(\rho_{\mathrm{steel}}P_{\mathrm{steel}})}$ (where P_{steel} is the longitudinal wave modulus of the stainless steel), and add a new constraint of $Z_{\mathrm{R}}\geq0.4$ in the formulation in addition to the objective function and constraints in Eqs. (1a)-(1h). In the following example with the impedance match constraint $Z_{\mathrm{R}}\geq0.4$, the other optimization parameters are selected as $f_{\max}$=19.5 kHz, δ_{E}=0.1, δ_{F}=0.2, δ_{p}=1.37 and e^{*}=0.001 m. Figure S5(a) shows the optimized HEMM which has a negative ρ_{xx} and a negative E_{xx} within the frequency ranges of (10.859 kHz, 19.516 kHz) and (19.604 kHz, 28.654 kHz), respectively. Both ρ_{yy} and E_{yy} are always positive in the same frequency ranges. This proves the occurrence of the hyperbolic dispersion in the frequency range of (10.859 kHz, 19.516 kHz) in Fig. S5(b). The optimized HEMM in Fig. S5(a) is composed of six blocks and four thick connections, which has a larger mass density and a stronger stiffness than that of H1 in Fig. 1(d). The increased stiffness gives rise to a better impedance match. We plot in Fig. S5(c) the corresponding imaging simulation at 14 kHz. Here, an improved larger imaging transmission and a noticeable higher imaging resolution (FWHM=0.138λ) are obtained, which demonstrates the positive effect of the proposed impedance match constraint. However, a better impedance match often requires the simultaneously large mass density and stiffness, leading to an incapability within the ultra-low frequency region (λ>=90a).

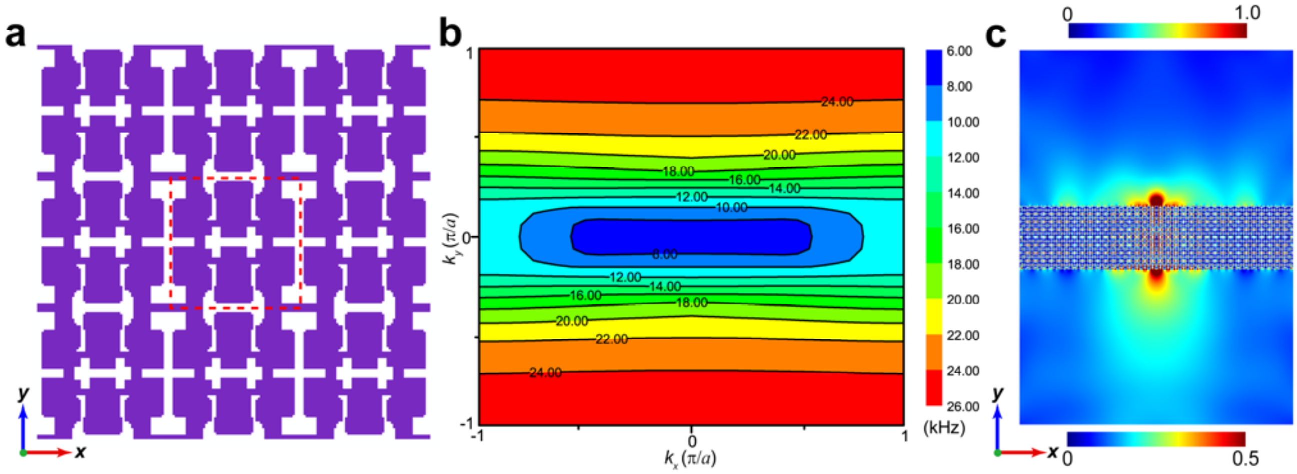

Figure S5 | Optimized HEMM with the impedance match constraint. (**a**) 3×3 lattice structure (the unit-cell is marked by the dashed line). (**b**) EFCs of the third band for the HEMM in (a). (**c**) Field magnitude patterns of the longitudinal wave mode showing the imaging by the 35×8 slab based on the HEMM in (a) at 14 kHz. The imaging resolution in (c) is FWHM=0.138λ. The point wave source is located in the position 0.02 m away from the upper side of the HEMM slab.

References

[1] Liu, X. N., Hu, G. K., Sun, C. T. & Huang, G. L. Wave propagation characterization and design of two-dimensional elastic chiral metacomposite. *J. Sound Vib.* **330,** 2536-2553 (2011).

[2] Liu, X. N., Hu, G. K., Huang, G. L. & Sun, C. T. An elastic metamaterial with simultaneously negative mass density and bulk modulus. *Appl. Phys. Lett.* **98,** 251907 (2011).

[3] Zhou, X. & Hu, G. Superlensing effect of an anisotropic metamaterial slab with near-zero dynamic mass. *Appl. Phys. Lett.* **98,** 263510 (2011).

[4] Shen, C., Xie, Y., Sui, N., Wang, W., Cummer, S. A. & Jing, Y. Broadband acoustic hyperbolic metamaterial. *Phys. Rev. Lett.* **115,** 254301 (2015).

[5] Zhu, R., Chen, Y. Y., Wang, Y. S., Hu, G. K. & Huang, G. L. A single-phase elastic hyperbolic metamaterial with anisotropic mass density. *J. Acoust. Soc. Am.* **139,** 3303-3310 (2016).

[6] Zhu, J., Christensen, J., Jung, J., Martin-Moreno, L., Yin, X., Fok, L., Zhang, X. & Garcia-Vidal, F. J. A holey-structured metamaterial for acoustic deep-subwavelength imaging. *Nat. Phys.* **7,** 52-55 (2011).

[7] Dong, H. W., Zhao, S. D., Wang, Y. S. & Zhang, C. Topology optimization of anisotropic broadband double-negative elastic metamaterials, *J. Mech. Phys. Solids* **105**, 54-80, (2017).

[8] Dong, H. W., Su, X. X. & Wang, Y. S. Multi-objective optimization of two-dimensional porous phononic crystals. *J. Phys. D: Appl Phys.* **47,** 155301 (2014).

[9] Dong, H. W., Su, X. X., Wang, Y. S. & Zhang C. Topology optimization of two-dimensioanl phononic crystals based on the finite element method and genetic algorithm. *Struct. Multidisc. Optim.* **50**, 593-604 (2014).

[10] Sigmund, O. & Petersson, J. Numerical instabilities in topology optimization: a survey on procedures dealing with checkerboards, mesh-dependencies and local minima. *Struct. Multidisc. Optim.* **16,** 68-75 (1998).

[11] Liu, F. & Liu, Z. Elastic waves scattering without conversion in metamaterials with simultaneous zero indices for longitudinal and transverse waves. *Phys. Rev. Lett.* **115,** 175502 (2015).
